# Effect of heat exposure on the growth and developmental competence of bovine oocytes derived from early antral follicles

**DOI:** 10.1038/s41598-022-12785-2

**Published:** 2022-05-25

**Authors:** Kohei Kawano, Kenichiro Sakaguchi, Chelenga Madalitso, Nattapong Ninpetch, Shintaro Kobayashi, Eri Furukawa, Yojiro Yanagawa, Seiji Katagiri

**Affiliations:** 1 Laboratory of Theriogenology, Graduate School of Veterinary Medicine, Hokkaido University, N18, W9, Kita-ku, Sapporo, 060-0818 Japan; 2 Laboratory of Theriogenology, Department of Clinical Sciences, Faculty of Veterinary Medicine, Hokkaido University, N18, W9, Kita-ku, Sapporo, 060-0818 Japan; 3 Institute of Cell Biology, School of Biological Sciences, College of Science and Engineering, University of Edinburgh, The Hugh Robson Building, 15 George Square, Edinburgh, EH8 9XD UK; 4 Department of Clinical Studies, Faculty of Veterinary Medicine, Lilongwe University of Agriculture and Natural Resources, P.O. Box 219, Lilongwe, Malawi; 5 Laboratory of Public Health, Department of Preventive Veterinary Medicine, Faculty of Veterinary Medicine, Hokkaido University, N18, W9, Kita-ku, Sapporo, 060-0818 Japan

**Keywords:** Reproductive biology, Oogenesis, Embryology, Climate-change impacts

## Abstract

In dairy cows, low fertility caused by summer heat stress continues into the cooler autumn season. This can be caused by impaired oocyte quality in small growing follicles during summer. Here, we subjected oocyte–cumulus–granulosa complexes (OCGCs) derived from early antral follicles (0.5–1 mm) to in vitro growth (IVG) culture under two different temperature settings (the control and heat shock groups), and evaluated effects of heat exposure on growth and developmental competence of oocytes, factors affecting the developmental competence of oocytes (steroidogenesis of granulosa cells, oxidative stress in oocytes, and cell-to-cell communication between oocytes and somatic cells). Oocyte diameters after culture were smaller in the heat shock group. Although nuclear maturation and cleavage rates were similar between the groups, blastocyst rates were lower in the heat shock group (0.0%) than in the control group (27.7%), and reduced glutathione (GSH) levels in oocytes were lower in the heat shock group. Supplementation of cysteine, which stimulates GSH synthesis, increased GSH level and improved blastocyst rate of heat shocked oocytes (27.9%). These results suggest that heat exposure impairs the growth and developmental competence of oocytes in early antral follicles through GSH depletion, which can induce low fertility during summer and the following autumn.

## Introduction

Summer heat stress is one of the major contributing factors to low fertility in lactating dairy cows^1^. Summer heat stress causes maternal hyperthermia, which induces various types of dysfunction in female reproductive tracts at the cellular level, leading to low fertility^2,3^. Impaired developmental competence of oocytes at the germinal vesicle (GV) stage is one of the causes of low fertility during summer^4^. Oocytes collected from medium-sized antral follicles (2–8 mm in diameter) during summer exhibit lower developmental competence following in vitro fertilization (IVF)^5–7^ or chemical activation for parthenogenesis than those during the cool season^8^. The negative effects of heat stress on reproduction continue for 1 or 2 months after summer heat stress ends^8,9^. Indeed, the low fertility of cows and reduced developmental competence of oocytes are also found in the subsequent cooler autumn season^9^. Coincidentally, early antral follicles (0.5–1 mm in diameter) require about 1 month to develop into large dominant follicles^10^. This coincidence suggests that exposure to summer heat stress during the early antral follicle stage is associated with low fertility and reduced developmental competence of oocytes in the subsequent autumn. Although the early antral follicle stage is the critical stage for oocytes to acquire developmental competence^11^, there are no reports focusing on the effect of heat stress on the growth of early antral follicles.

Low developmental competence of heat stressed GV stage oocytes derived from antral follicles (2–8 mm) is associated with an increase in the variation in fatty acid profiles of the membrane^8^, altered transcriptional levels of genes involved in oogenesis, folliculogenesis, and embryonic development^7^. The GV stage oocytes exposed to high temperature in vitro or collected in summer also exhibit disrupted nuclear and cytoplasmic events, including translocation of the cortical granule to the oolemma^12^, and impaired mitochondrial distribution and polarization in matured oocytes^13^. However, the mechanism underlying the disruption in the developmental competence of GV stage oocytes in the process of growth is still not clear.

Altered steroidogenesis in growing follicles may be associated with reduced oocyte developmental competence during summer. Steroid hormones are important factors needed to acquire oocyte developmental competence during follicular growth. In vitro, estradiol (E_2_) is essential for oocytes to acquire maturational and developmental competence during in vitro growth (IVG) culture of bovine oocyte–cumulus–granulosa complexes (OCGCs) derived from early antral follicles (0.4–0.7 mm in diameter)^11,14,15^. However, in vivo studies demonstrated that the E_2_ concentration in the follicular fluid of dominant follicles was lower on day 7 of the estrous cycle during the hot season than the cool season^16^. Furthermore, the steroidogenic capacity of antral follicles of various sizes is also disrupted by heat stress^17^. The E_2_ and androstenedione (A_4_) production levels were reduced in the cultured granulosa and theca cells obtained from medium-sized follicles (6–9 mm in diameter) 3 weeks after acute heat stress^10^, suggesting that early antral follicles are sensitive to heat stress. However, the relationship between the steroidogenesis in growing follicles and developmental competence of oocytes under the effects of heat stress has not been investigated directly.

Hyperthermia-induced oxidative stress is suggested to be another underlying mechanism by which heat stress impairs the developmental competence of oocytes^18^. Studies using an in vitro maturation (IVM) system revealed that heat exposure increases the intracellular reactive oxygen species (ROS) levels in bovine oocytes and reduces the percentage of oocytes developing to blastocysts^19,20^. Glutathione is the most abundant non-protein thiol in mammalian cells^21^ and reduced glutathione (GSH) maintains the cellular redox status, and protects the cell from ROS^22^. Granulosa cells have a critical role in supplying GSH to oocytes by cell-to-cell communication via transzonal projections (TZPs)^23^. The decrease in the intracellular GSH level in oocytes occurs along with an increase in the intracellular ROS level caused by heat exposure during IVM^19^. The addition of cysteine to the IVM medium, which stimulates the GSH synthesis^24^, reduced the ROS level in oocytes and mitigated the negative effects of heat exposure on oocyte developmental competence^19^. One study aimed to improve the oocyte quality of dairy cows during summer by feeding them unsaturated fatty acids, which have antioxidative capacity^25^. However, this feeding procedure did not improve the developmental competence of the collected oocytes^25^. If we can develop an experimental model mimicking the follicular growth under heat stress by using an IVG culture system, it may be easier to evaluate the effects of the supplementation of some substances (i.e., antioxidants) on the developmental competence of oocytes exposed to heat stress during the growth phase. The effective substances selected in the experimental model could be useful to manage dairy cows during summer to collect better quality oocytes for IVM and improve fertility in the subsequent cooler autumn.

In in vivo, seasonal heat stress is likely to impair the developmental competence of oocytes at growing phase not only through high body temperature, but also through other physiological changes under the heat stressed environment (i.e. impaired gonadotropins secretion, altered metabolic system due to reduced feed intake, and so on)^1^. However, in this study, we aimed to investigate the effects of high temperature in the physiological range on GV stage oocytes during the growth phase using an IVG culture system for bovine oocytes. IVG culture enables oocytes without maturational competence from early antral follicles (0.5–1 mm in diameter) to grow and acquire maturational and developmental competence to develop into the blastocyst stage^26,27^. First, we examined the effect of heat exposure during IVG culture on the competence of growth, maturation, and subsequent developmental competence to the blastocyst stage. Second, to investigate the mechanisms by which heat exposure reduces the developmental competence of GV stage oocytes, we evaluated E_2_ and progesterone (P_4_) production from granulosa cells, the intracellular ROS and GSH levels of oocytes, and the number of TZPs between oocytes and cumulus cells. Third, we investigated the rescue effect of cysteine supplementation to IVG medium on the GSH level and the developmental competence of oocytes exposed to high temperature during IVG.

## Results

### Experiment 1: effects of heat exposure during IVG culture on OCGC morphology, oocyte growth, and the maturational and developmental competence of oocytes

**Figure 1:**
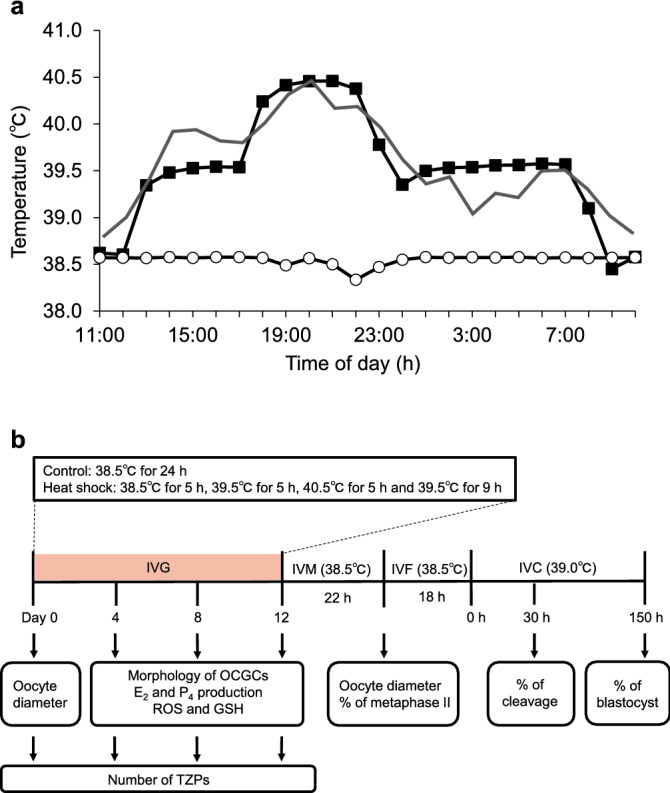
Temperature conditions during in vitro growth (IVG) culture in the control and heat shock groups (**a**) and schematic illustration of the experimental design (**b**). (**a**) Daily changes in the rectal temperature of lactating dairy cows under heat stress (solid line), and culture temperatures for the heat shock group (filled square) and control group (open circle) monitored by a data logger placed in the incubator (averages of temperature measured every hour for 12 days). Oocyte–cumulus–granulosa complexes (OCGCs) in the heat shock group were cultured at a range of temperatures (38.5 °C for 5 h, 39.5 °C for 5 h, 40.5 °C for 5 h, and 39.5 °C for 9 h) similar to those experienced by heat-stressed cows^29^. OCGCs in the control group were cultured at a constant temperature of 38.5 °C for 24 h, mimicking the body temperature of cows without heat stress^28^. (**b**) OCGCs derived from early antral follicles (0.5–1 mm in diameter) were cultured for 0, 4, 8, or 12 days in an IVG culture. The oocyte diameter was evaluated on day 0 of the IVG culture. The morphology of OCGCs (viability of OCGCs and antrum formation in granulosa cell layers) was evaluated every 4 days during the IVG culture (days 4, 8, and 12). After 12 days of IVG, some surviving OCGCs were subjected to in vitro maturation (IVM). After IVM, the diameter and nuclear status of some oocytes were evaluated. Some oocytes after IVM were subjected to in vitro fertilization (IVF) and an in vitro culture (IVC) to evaluate developmental competence. OCGCs cultured until IVM (evaluation of growth and nuclear status) or IVC (evaluation of developmental competence) were derived from different culture sessions. The concentrations of estradiol-17β (E_2_) and progesterone (P_4_) in the IVG media, and the intracellular reactive oxygen species (ROS) and reduced glutathione (GSH) levels in the oocytes were evaluated every 4 days during IVG culture (days 4, 8, and 12). The number of transzonal projections (TZPs) was evaluated on day 0 and every 4 days during IVG culture (days 4, 8, and 12).

**Figure 2:**
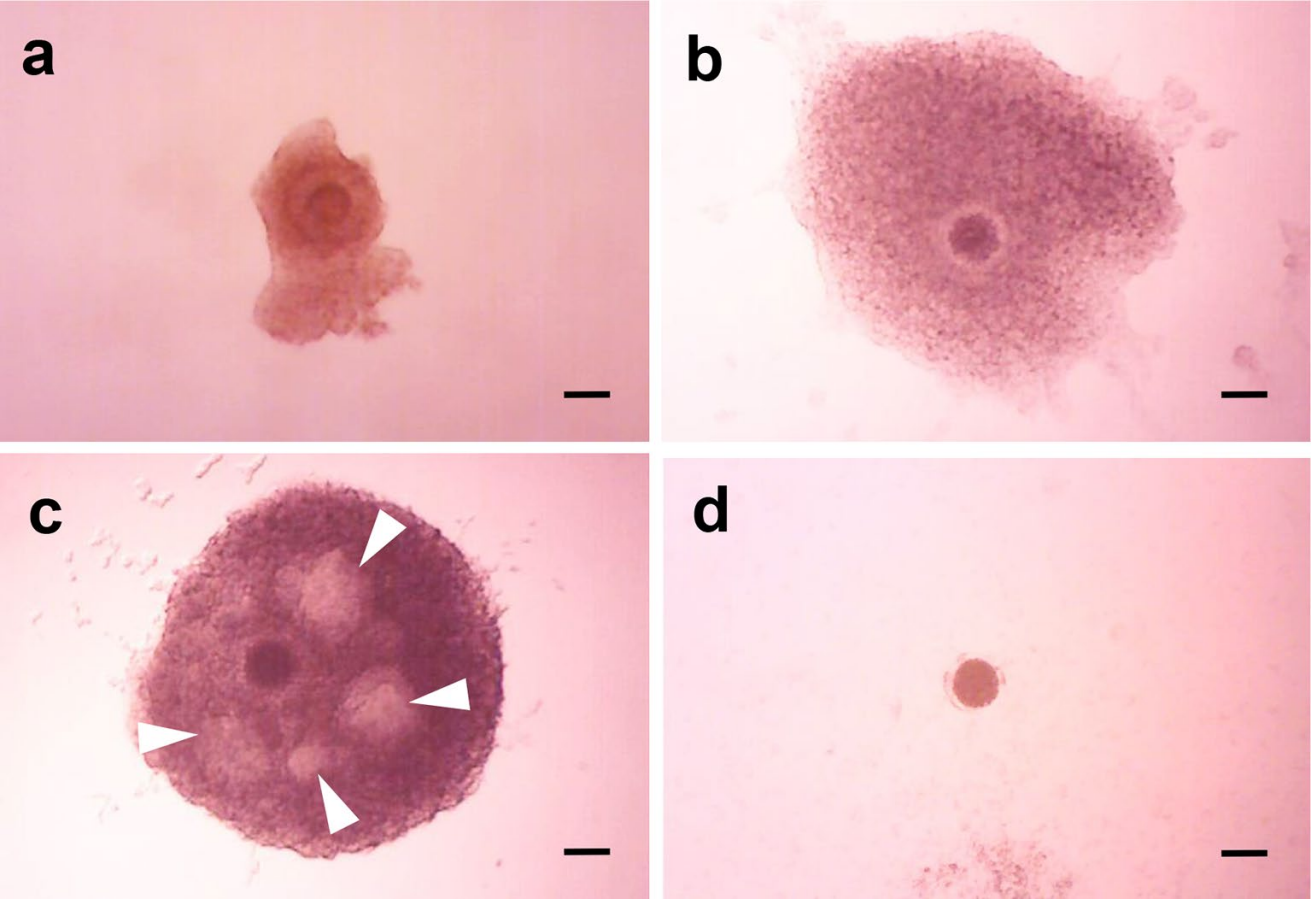
Morphology of oocyte–cumulus–granulosa complexes (OCGCs) before and after 12 days of in vitro growth (IVG) culture. (**a**) Isolated OCGC before IVG culture. (**b**) Surviving OCGC without antrum formation in the granulosa cell layer after 12 days of IVG culture. (**c**) Surviving OCGC with antrum formation (white arrowhead) in the granulosa cell layer. (**d**) Degenerated OCGCs after 12 days of IVG culture. Scale bar = 100 µm.

**Figure 3:**
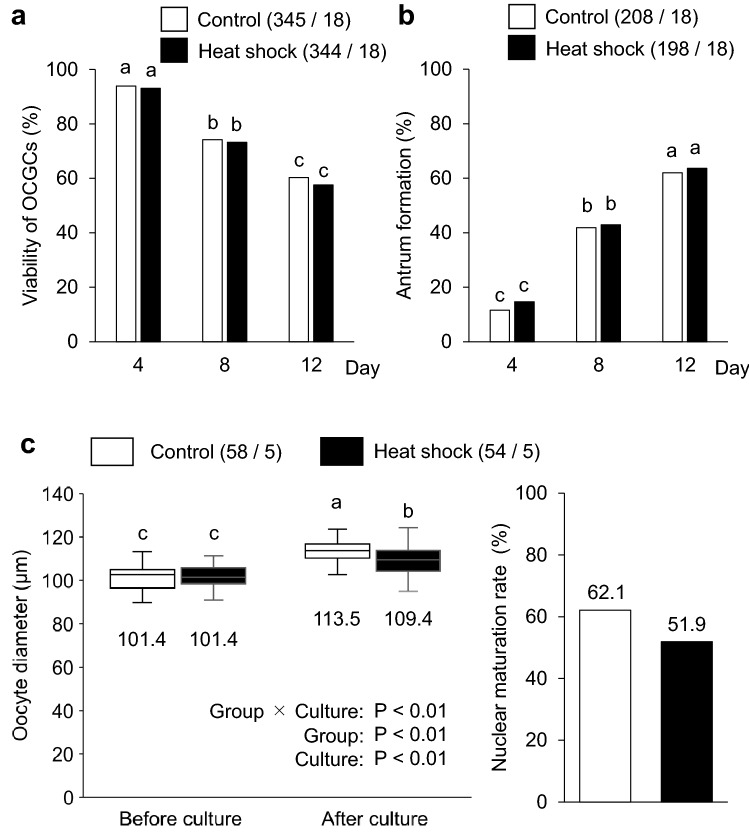
Effects of heat shock during in vitro growth (IVG) culture on the viability (**a**), antrum formation (**b**) of oocyte–cumulus–granulosa complexes (OCGCs), and the growth and nuclear status of oocytes after in vitro maturation (IVM) (**c**). Numbers in parentheses indicate the number of OCGCs and replicates. (**a**) The viability of OCGCs was calculated based on 689 OCGCs that were cultured until the end of IVG culture (12 days) (345 from the control group and 344 from the heat shock group). (**b**) The percentage of antrum formation in the granulosa cell layer was calculated based on 406 OCGCs surviving on day 12 (208 from the control group and 198 from the heat shock group). ^abc^Different letters indicate a significant difference between the duration of culture in the same group (P < 0.05). (**c**) Lines on the boxes in the box-and-whisker plot delineate the 25th, 50th, and 75th percentiles, while the whiskers depict the 10th and 90th percentiles. Values below boxes in the box-and-whisker plot indicate the mean diameters (µm) of the oocytes. Numbers in parentheses indicate the number of oocytes submitted for IVM. ^a–c^Different letters indicate significant differences (P < 0.01).

To examine the effects of heat exposure on oocyte growth, maturation, and developmental competence, we conducted IVG of bovine OCGCs derived from early antral follicles under two different temperature settings (control and heat shock groups; Fig. 1a). In the control group, OCGCs were cultured at 38.5 °C for 12 days, mimicking the body temperature in non-heat-stressed dairy cows^28^. In the heat shock group, OCGCs were cultured using a temperature cycle of 38.5 °C for 5 h, 39.5 °C for 5 h, 40.5 °C for 5 h, and 39.5 °C for 9 h for 12 days, mimicking the body temperature in dairy cows under heat stress^29^. After IVG culture, the oocytes were subjected to IVM, IVF, and in vitro culture (IVC) of embryos, and the diameter, nuclear maturational competence, and developmental competence of the oocytes were evaluated (Fig. 1b). The blastocyst rates were calculated from the number of inseminated oocytes. During IVG culture, we observed the morphological appearance of OCGCs in the two groups (Fig. 2), but there was no significant difference in the viability or antrum formation rates of OCGCs between the control and heat shock groups (Fig. 3a,b). Both the group and culture period significantly affected the mean diameter of the oocytes (P < 0.001) (Fig. 3c). In addition, there was a significant interaction of the group and culture period on the mean diameter of the oocytes (P < 0.001). The mean diameter of the oocytes in the control group increased from 101.4 before IVG to 113.5 µm after IVG and IVM (P < 0.05). The increase in the diameter was also significant in the heat shock group (from 101.4 to 109.4 µm, P < 0.05), while the mean diameter of the oocytes after IVG and IVM was significantly smaller in the heat shock group than in the control group (P < 0.05). The nuclear maturation and cleavage rates were similar between the control group (62.1% and 55.3%, respectively) and the heat shock group (51.9% and 45.8%, respectively) (Fig. 3c, Table 1). However, no oocytes developed to blastocysts in the heat shock group (0.0%), while 27.7% of oocytes developed to blastocysts in the control group (P < 0.05) (Table 1).

**Table 1:** Effects of heat exposure during in vitro growth (IVG) culture of oocyte–cumulus–granulosa complexes (OCGCs) on their oocyte developmental competence.

Group	No. of oocytes (replicates)	Cleavage (%)	Blastocyst (%)*	Cell no. in blastocysts (n)
Control	47 (4)	55.3	27.7^a^	104.5 ± 9.3 (13)
Heat shock	48 (4)	45.8	0.0^b^	–

Cell number in the blastocysts is presented as means ± SEM.

*Blastocyst rates were calculated based on the number of inseminated oocytes.

^a^^,b^Different superscripts indicate significant differences between the groups (P < 0.05).

### Experiment 2: effects of heat exposure during IVG culture on the steroidogenesis of granulosa cells, ROS and GSH levels in oocytes, and number of TZPs in oocytes

**Figure 4:**
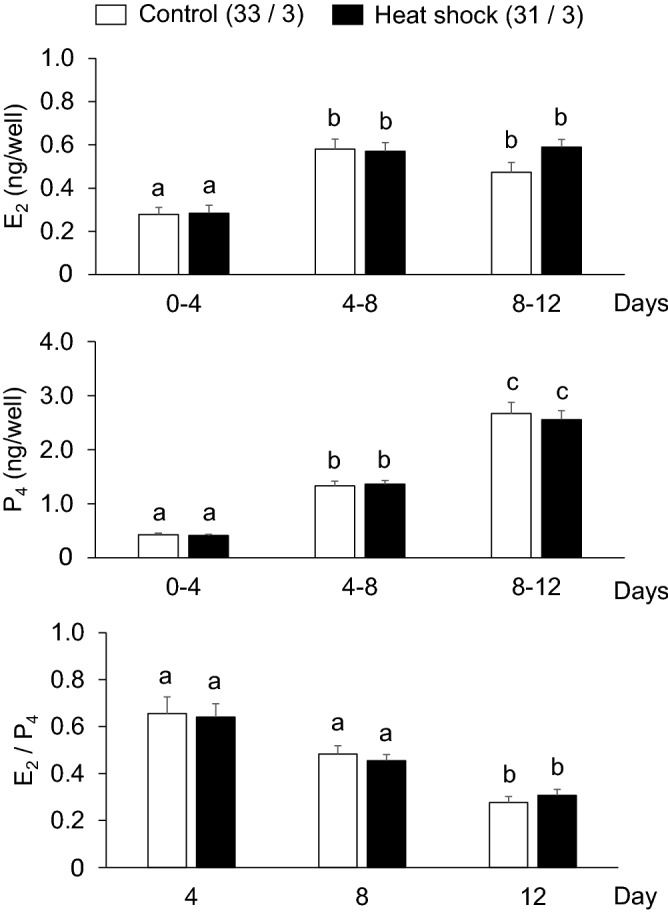
Effects of heat shock during in vitro growth (IVG) culture on the production of estradiol-17β (E_2_) and progesterone (P_4_) by oocyte–cumulus–granulosa complexes (OCGCs), and the E_2_/P_4_ ratio in culture media. Numbers in parentheses indicate the number of OCGCs and replicates. The culture media for the hormone assays were derived from some OCGCs used to evaluate the nuclear status after in vitro maturation (three replicates). ^a–c^Different letters indicate significant differences between culture periods in the same group (P < 0.05). Error bars indicate SEM.

**Figure 5:**
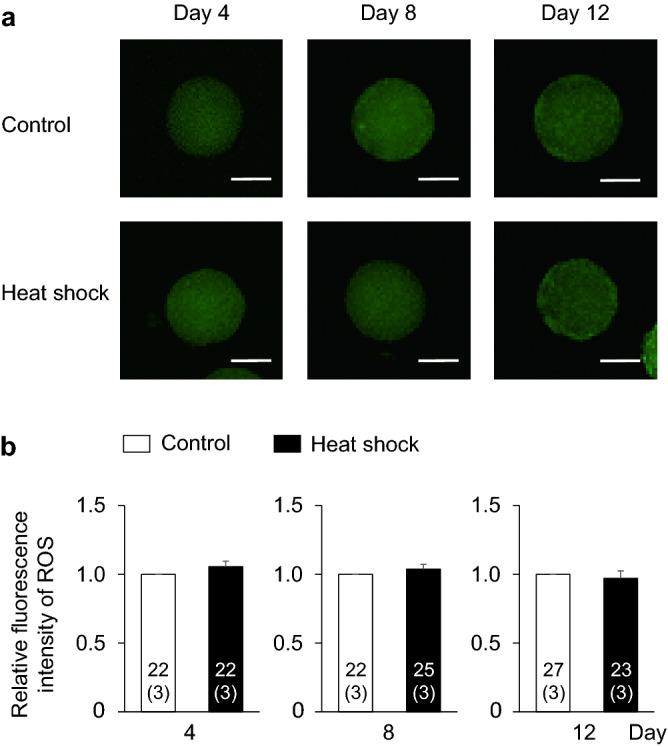
Effects of heat shock during in vitro growth (IVG) culture on the intracellular reactive oxygen species (ROS) levels in oocytes. (**a**) Representative fluorescent photomicrographs of IVG oocytes detected with 2ʹ,7ʹ-dichlorodihydrofluorescein diacetate (DCHFDA). The intracellular ROS levels in oocytes were evaluated every 4 days during IVG culture (days 4, 8, and 12) in the control group (upper panels) and the heat shock group (lower panels). Scale bar = 50 µm. (**b**) The relative fluorescent intensity for ROS levels from the control and heat shock groups every 4 days during IVG culture (days 4, 8, and 12). The fluorescent intensity of ROS was measured using a total of 141 oocytes (three replicates each). Numbers in the bar graph indicate the number of oocytes, while the number of replicates is shown in parentheses. Fluorescence intensity of the heat shock group was normalized to that of the control group on the same culture day. Error bars indicate SEM.

**Figure 6:**
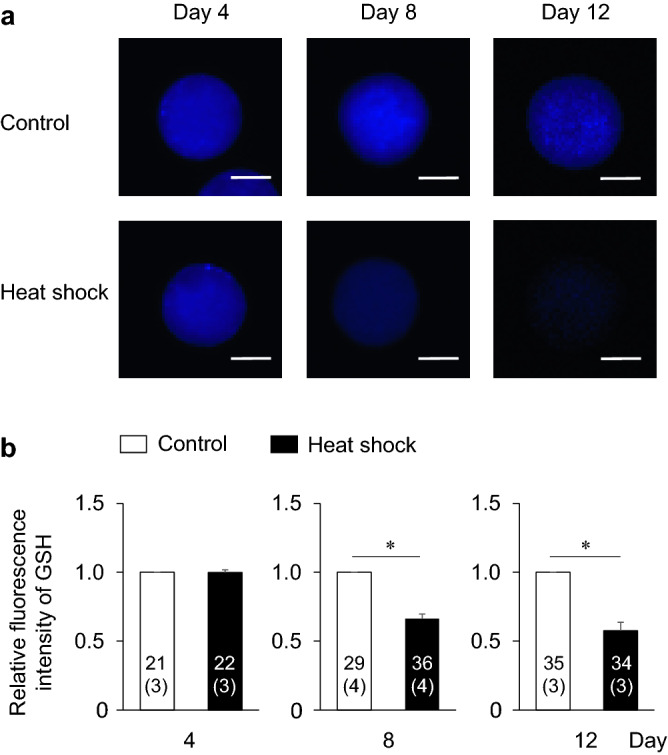
Effects of heat shock during in vitro growth (IVG) culture on the intracellular reduced glutathione (GSH) levels in oocytes. (**a**) Representative fluorescent photomicrographs of IVG oocytes detected with CellTracker Blue. The intracellular GSH levels in the oocytes were evaluated every 4 days during IVG culture (days 4, 8, and 12) in the control group (upper panels) and the heat shock group (lower panels). Scale bar = 50 µm. (**b**) The relative fluorescent intensity for GSH levels from the control and heat shock groups every 4 days during IVG culture (days 4, 8, and 12). The fluorescent intensity of GSH was measured using a total of 177 oocytes (three–four replicates each). Numbers in the bar graph indicate the number of oocytes, while the number of replicates is shown in parentheses. Fluorescence intensity of the heat shock group was normalized to that of the control group on the same culture day. Error bars indicate SEM. *An asterisk indicates a significant difference between the control and heat shock groups on the same day (P < 0.05).

**Figure 7:**
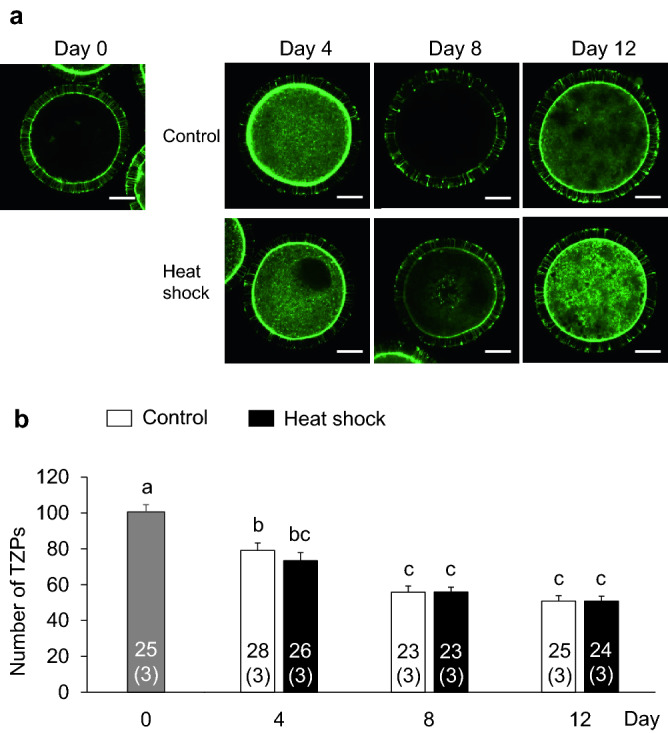
Effects of heat shock during in vitro growth (IVG) culture on the number of transzonal projections (TZPs) between oocytes and cumulus cells. (**a**) Fluorescence staining of TZPs between oocytes and surrounding cumulus cells detected with fluorescein isothiocyanate-labeled Phalloidin. The number of TZPs was evaluated on days 0 (upper left panel), and every 4 days during IVG culture (days 4, 8, and 12) in the control group (upper panels) and heat shock group (lower panels). Scale bar = 25 µm. (**b**) The number of TZPs in IVG oocytes from the control and heat shock groups at day 0, and every 4 days during IVG culture (days 4, 8, and 12). The number of TZPs was evaluated using a total of 174 oocytes (three replicates each). Numbers in the bar graph indicate the number of oocytes, while the number of replicates is shown in parentheses. Error bars indicate SEM. ^a–c^Different letters indicate significant differences (P < 0.05).

To clarify the mechanism responsible for the impaired developmental competence in the heat shock group, we evaluated E_2_ and P_4_ production from granulosa cells, the intracellular ROS and GSH levels in oocytes, and the number of TZPs between oocytes and cumulus cells, which are related to the developmental competence of oocytes (Fig. 1b). The E_2_ and P_4_ production levels were similar between the control and heat shock groups (Fig. 4). In both groups, the E_2_ production increase from days 0–4 to 4–8 was maintained until the end of the IVG culture (days 8–12). On the other hand, P_4_ production continuously increased during the culture period. The E_2_/P_4_ ratio did not differ between the two groups. Furthermore, the intracellular ROS levels in the oocytes did not differ between the two groups (Fig. 5); however, the GSH levels in the oocytes were lower in the heat shock group than in the control group at days 8 and 12 (P < 0.05) (Fig. 6). The number of TZPs between the oocytes and the surrounding cumulus cells did not differ between the two groups (Fig. 7). In both groups, the number of TZPs decreased until day 8 of IVG culture.

### Experiment 3: effects of cysteine supplementation on the developmental competence and GSH levels of oocytes exposed to high temperature during IVG culture

**Figure 8:**
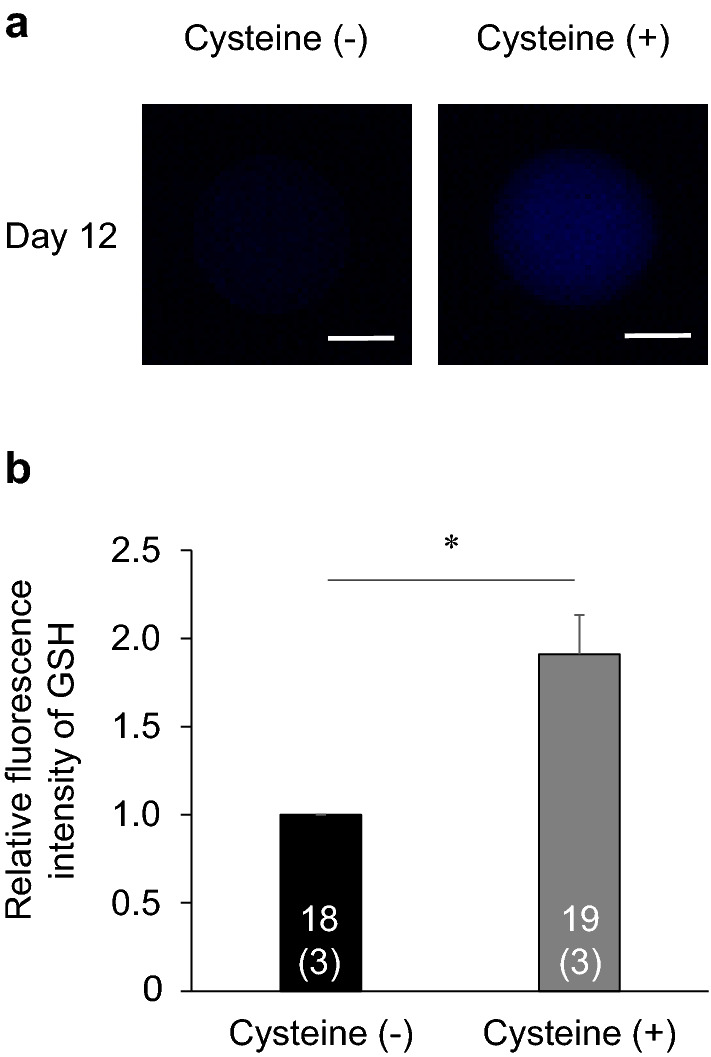
Effect of cysteine supplementation on the GSH levels of oocytes exposed to high temperature during in vitro growth (IVG) culture. (**a**) Representative fluorescent photomicrographs of IVG oocytes detected with CellTracker Blue. The intracellular GSH levels were evaluated on day 12 of IVG culture in the cysteine untreated group (left panels) and cysteine treated group (right panels). Scale bar = 50 µm. (**b**) The relative fluorescent intensity for GSH levels from the cysteine untreated and treated group after day 12 of IVG culture. The fluorescent intensity of GSH was measured using a total of 37 oocytes (three replicates each). Numbers in the bar graph indicate the number of oocytes, while the number of replicates is shown in parentheses. The fluorescence intensity of oocytes in the cysteine treated group was normalized to those in the cysteine untreated group. Error bars indicate SEM. *An asterisk indicates a significant difference between the cysteine untreated and treated groups (P < 0.05).

To clarify whether the promotion of GSH synthesis in OCGCs restores the developmental competence of oocytes exposed to high temperature during IVG, we investigated the effects of cysteine supplementation to the culture medium on the developmental competence of oocytes exposed to high temperature during IVG. In this experiment, we examined the cysteine untreated group (IVG medium supplemented with 0.0 mM cysteine) and cysteine treated group (IVG medium supplemented with 1.2 mM cysteine). In a previous study, supplementation of 1.2 mM cysteine to the medium increased the GSH levels and improved the developmental competence of oocytes exposed to high temperature during IVM^19^, therefore this cysteine concentration was adopted for this study. The cleavage rates were similar between the cysteine treated group (67.4%) and the cysteine untreated group (57.1%) (Table 2). However, the blastocyst rate was significantly higher in the cysteine treated group (27.9%) than in the cysteine untreated group (6.1%) (P < 0.05). In addition, the cell number in the blastocysts was slightly higher in the cysteine treated group (122.1 ± 10.8; mean ± standard error of the mean (SEM)) than in the cysteine untreated group (74.3 ± 6.8; mean ± SEM) (P = 0.06). The intracellular GSH levels in oocytes after 12 days of IVG culture were higher in the cysteine treated group than in the cysteine untreated group (P < 0.05) (Fig. 8).

**Table 2:** Effect of cysteine supplementation on the developmental competence of oocytes exposed to high temperature during in vitro growth (IVG) culture.

Treatment	No. of oocytes (replicates)	Cleavage (%)	Blastocyst (%)*	Cell no. in blastocysts (n)
Cysteine (−)	49 (4)	57.1	6.1^b^	74.3 ± 6.8 (3)^B^
Cysteine ( +)	43 (4)	67.4	27.9^a^	122.1 ± 10.8 (12)^A^

Cell numbers in the blastocysts are presented as means ± SEM.

*Blastocyst rates were calculated based on the number of inseminated oocytes.

^a^^,b^Different superscripts indicate significant differences between the groups (P < 0.05).

^A^^,B^Different superscripts indicate slight differences between the groups (P = 0.06).

## Discussion

To the best of our knowledge, we have for the first time demonstrated that heat exposure during IVG culture impairs the growth and developmental competence of oocytes derived from early antral follicles (0.5–1 mm). The intracellular GSH depletion in oocytes can be one cause of the impaired developmental competence of oocytes caused by heat exposure during oocyte growth from early antral follicles.

Although the cleavage rate was similar between the control and heat shock groups, the blastocyst rate was significantly lower in the heat shock group than in the control group. This result is similar to previous studies that compared the developmental competence of oocytes derived from 3 to 8 mm follicles in summer and winter^6,7^. These studies also showed that the cleavage rates were similar between the groups or slightly lower in summer than in winter, whereas the blastocyst rates were significantly lower in summer than in winter. However, they compared the results between two different experimental terms and using different oocyte sources. Our experimental model can be used to investigate the mechanisms by which summer heat stress impairs the developmental competence of oocytes using the same oocyte sources at the same time between the control and heat shock groups.

The mean diameter of the oocytes after IVG and IVM was significantly smaller in the heat shock group than in the control group. Impaired oocyte growth can be one of the characteristics associated with the reduced developmental competence of oocytes caused by heat exposure. It is known that the full competence for the meiotic maturation and subsequent embryonic development is acquired at an oocyte diameter of about 110 µm^30,31^. Therefore, an oocyte diameter ≥ 110 µm could be an important indicator to determine whether or not an oocyte will acquire subsequent developmental competence. Consistent with a previous study, the percentage of oocytes ≥ 110 µm in diameter was higher in the control group (75.9%) than in the heat shock group (46.3%) (P < 0.01) in the present study.

The E_2_ and P_4_ production levels did not differ between the control and heat shock groups. Consistent with this finding, the rates of antrum formation, which can be an indicator of healthy steroidogenesis in OCGCs^15,32^, did not differ between the two groups. A previous study showed that OCGCs that formed an antrum produce more E_2_ and less P_4_ than OCGCs without an antrum in bovine IVG^32^. On the other hand, summer heat stress reduces the peripheral E_2_ concentration^33,34^ and the E_2_ concentration in the follicular fluid of the dominant follicle in dairy cows in the late lactation period^16^. The P_4_ concentration in the follicular fluid of the dominant follicle is not affected by seasonal heat stress in lactating dairy cows^16^. One possible reason we could not find any difference in E_2_ production compared to the in vivo situation may have been the absence of theca cells in the IVG culture system in the present study. Heat stress may suppress E_2_ production by inhibiting the systemic endocrine system or the function of theca cells in vivo. Heat stress was previously shown to reduce the number of luteinizing hormone (LH) pulses in lactating dairy cows^35^. In addition, theca cells are highly susceptible to heat stress; seasonal heat stress drastically reduces A_4_ production by theca cells^16^, and this reduction seems to continue for a long period after the end of summer heat stress^10,16^. These changes may lead to a decline in E_2_ secretion by granulosa cells in vivo. Our results clearly suggest that granulosa cells from early antral follicles have relatively higher resistance against high temperature to maintain steroidogenesis, which is the main physiological function of granulosa cells during follicular growth.

Although the intracellular ROS levels in the oocytes did not differ between the control and heat shock groups, the GSH levels in the oocytes were lower in the heat shock group than in the control group. GSH is one of the most important scavengers of ROS^22^. GSH consumption may have been higher in the heat shock group than in the control group to alleviate the increased oxidative stress in the oocyte cytoplasm. In the present study, a GSH level decline in the heat shock group was observed at days 8 and 12. The diameter of oocytes becomes significantly larger even during the late culture period (between days 10 and 12) in the bovine IVG of OCGCs derived from early antral follicles^36^. In addition, the number of granulosa cells during IVG culture of OCGCs markedly increases between days 4 and 12^26^. Therefore, the demand for amino acids for GSH synthesis (cysteine, glutamic acid, glycine) may increase to support the growth of oocytes, granulosa cell proliferation, and to maintain the oxidative stress in OCGCs as the culture period becomes longer. Therefore, the GSH supply from cumulus-granulosa cells to oocytes during this period may have been reduced in the heat shock group. Although we hypothesized that GSH depletion in the heat shock group may also be caused by impaired intracellular communications between growing oocytes and the surrounding cumulus cells, there was no difference in the number of TZPs between the control and heat shock groups. These results suggest that communication between oocytes and surrounding somatic cells could be maintained at the same level as the control group in the heat shock group, while the production of GSH in granulosa cells or oocytes themselves diminished in the heat shock group. In a future study, we should examine the enzymes related to GSH synthesis and consumption in OCGCs and their metabolism of amino acids, which is necessary for GSH synthesis in the IVG culture medium.

OCGCs in the heat shock group showed lower developmental competence concurrent with reduced intracellular GSH levels in the oocytes than those in the control group. In addition, the supplementation of cysteine, which stimulates GSH synthesis, increased the intracellular GSH level and developmental competence of the oocytes exposed to high temperature during IVG. These findings are consistent with previous reports indicating a relationship between low GSH levels in oocytes before or after IVM and their impaired developmental competence. Heat exposure during IVM^19^ or the addition of a GSH synthesis inhibitor to the pre-IVM culture medium^23^ decreased the intracellular GSH levels in oocytes and their developmental competence. This reduced developmental competence of oocytes subjected to intracellular GSH depletion is probably attributable to the decrease in antioxidant capacity during subsequent embryonic development. The present result suggests that heat exposure to OCGCs in the growth phase impairs developmental competence by depletion of GSH in the oocyte cytoplasm.

The present study suggests that OCGCs derived from early antral follicles are susceptible to high temperature in the physiological range. It takes about one month for an early antral follicle (0.5–1 mm in diameter) to develop into a large dominant follicle^37^; therefore, impaired oocyte developmental competence caused by heat exposure to early antral follicles during summer could be associated with low fertility in the subsequent cooler autumn. Some treatments or feeding management to improve the antioxidative capacity during the summer could ameliorate the negative effects of heat stress on the early antral follicles, thereby improving oocyte quality and fertility in the subsequent autumn. The culture system developed in the present study could replace in vivo trials to look for possible antioxidants to improve low oocyte quality and fertility caused by persistent effects of summer heat stress.

In conclusion, heat exposure during the IVG culture of OCGCs derived from early antral follicles impaired the growth of oocytes and the percentage of oocytes developing to the blastocyst stage. The present study suggested that the intracellular GSH depletion in oocytes (decrease in the antioxidative capacity) is one cause of the low oocyte developmental competence caused by summer heat stress, which can lead to impaired fertility in the subsequent autumn. However, it is necessary to keep in mind that we focused on the effects of high temperature in the physiological range on the cultured oocytes at growing phase in the present study. Not only high body temperature, but also alterations in gonadotropins secretion and metabolic system would be involved in low developmental competence of GV oocytes caused by seasonal heat stress in in vivo. Further studies will be necessary to clarify more detailed mechanisms by which summer heat stress reduces oocyte competence in the process of oocyte growth.

## Methods

All the chemicals used were purchased from Sigma-Aldrich (St. Louis, MO, USA), unless otherwise stated.

### Collection of OCGCs and IVG culture

Ethical approval for animal work was not required for this study as all the bovine ovaries were derived from cattle slaughtered at two local slaughterhouses for commercial food production purposes only.

**Figure 9:**
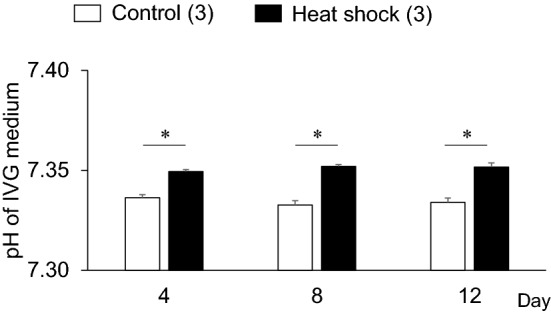
Effect of elevated culture temperature on the pH value of in vitro growth (IVG) medium without oocyte–cumulus–granulosa complexes (OCGCs). Numbers in parentheses indicate the number of IVG medium used for pH measurement. The experiment was repeated thrice. Error bars indicate SEM. *An asterisk indicates a significant difference between the control and heat shock groups on the same day (P < 0.05).

OCGCs were collected from the early antral follicles (0.5–1 mm in diameter) of bovine ovaries obtained from two slaughterhouses. Sliced ovarian cortex tissues were prepared using a surgical blade (No. 11), and follicles were dissected from cortical strips using a No. 20 blade under a stereomicroscope in an isolation medium; TCM-199 (31100-035, Thermo Fisher Scientific, Roskilde, Denmark) supplemented with 0.1% polyvinyl alcohol (PVA), 25 mM 2-[4-(2-Hydroxyethyl)-1-piperazinyl] ethanesulfonic acid (HEPES), 10 mM sodium bicarbonate, and 50 μg/mL gentamicin sulfate (isolation medium, pH 7.4) at 37 °C, as described elsewhere^32^. Early antral follicles were punctured to release OCGCs using a pair of fine forceps as described previously^36^. The growth medium was HEPES (25 mM)-buffered TCM-199 (12340-030, Thermo Fisher Scientific, Grand Island, NY, USA) supplemented with 0.91 mM sodium pyruvate, 5% (v/v) fetal calf serum (FCS; Invitrogen, Waltham, MA, USA), 4 mM hypoxanthine, 4% (w/v) polyvinylpyrrolidone (PVP) (MW 360,000), 50 μg/mL gentamicin sulfate, and 10 ng/mL A_4_ as a precursor for E_2_. OCGCs with oocytes surrounded by a cumulus investment and attached mural granulosa-cell layer (Fig. 2a) were cultured individually in a 96-well culture plate (Primaria 353872, Corning Incorporated, Corning, NY, USA) with 200 μL of growth medium for 12 days in humidified air with 5% CO_2_. OCGCs in the control group were cultured at 38.5 °C for 24 h, mimicking the body temperature in non-heat-stressed dairy cows (Fig. 1a)^28^. OCGCs in the heat shock group were cultured using a temperature cycle of 38.5 °C for 5 h, 39.5 °C for 5 h, 40.5 °C for 5 h, and 39.5 °C for 9 h, which is similar to the body temperature of heat-stressed dairy cows^29^. In each culture session, the temperature in the incubator was monitored using a data logger (GL10-TH, Graphtec, Kanagawa, Japan) at one-hour intervals for 12 days, and averages of the temperature were calculated (Fig. 1a). In addition, increases in temperature in vitro cause decreased CO_2_ solubility in the medium and an increase in medium pH, therefore we also confirmed the effects of elevated culture temperature on the pH value of the IVG medium. Half (100 μL) of the IVG medium without OCGCs was replaced every 4 days (days 4, 8, and 12). The pH values in the spent IVG medium were evaluated by the i-STAT system (G3^+^ cartridge, Abbot Point of Care Inc., Princeton, NJ, USA). Every measurement was performed at 22:00 (after 5 h incubation at 40.5 ℃ in the heat shock group). We found that the pH values in both the groups were in the range of 7.30 to 7.40, and were higher in the heat shock group than in the control group. However, the difference in the pH values between the groups was less than 0.02. In a previous study, there were no significant differences between pH 7.2 and pH 7.4 in the meiotic arrest of oocytes cultured with a meiotic inhibitor (dibutyryl cyclic adenosine monophosphate or hypoxanthine) and follicle-stimulating hormone (FSH)-stimulated meiotic resumption of oocytes^38^. The difference in pH between the control and heat shock groups is thought to be a slight difference throughout the 12 days of IVG culture and it is unlikely that the increase in the pH value caused by elevated culture temperature adversely affected the developmental competence of oocytes in the present study (Fig. 9). At the onset of the IVG culture, OCGCs were photographed under an inverted microscope (CK 40, Olympus, Tokyo, Japan) with an attached CCD camera (Moticam 2000, Shimadzu Rika Corporation, Tokyo, Japan). The diameters of the oocytes on day 0 were assessed using software (Motic Images Plus 2.2s, Shimadzu). In the 12-day IVG culture, half (100 μL) of the medium was replaced every 4 days. The spent medium was stored at − 30 °C for a hormone assay.

### Evaluation of OCGC morphology

The morphological appearance of the OCGCs was evaluated every 4 days during IVG culture under an inverted microscope. OCGCs with an evenly granulated ooplasm and completely enclosed by several layers of healthy cumulus and granulosa cells with or without cavities (Fig. 2b,c, respectively) were defined as normal. OCGCs with scattered cumulus and granulosa cells or denuded oocytes were defined as abnormal (Fig. 2d).

### Evaluation of growth and nuclear maturation

After IVG culture, OCGCs were subjected to IVM as previously described^39^. Briefly, OCGCs were individually cultured in microwell plates (Mini Trays 163 118; NUNC, Roskilde, Denmark) containing 6 mL of IVM medium at 38.5℃ under 5% CO_2_ in air for 22 h. The IVM medium consisted of HEPES-buffered TCM-199 supplemented with 0.2 mM sodium pyruvate, 20 µg/mL FSH (from porcine pituitary), 1 μg/mL E_2_, 10% FCS, and 50 μg/mL gentamicin sulfate. After IVM, the oocytes were denuded by pipetting, photographed, and their diameters were measured. Oocyte nuclear maturation was assessed using 1% (w/v) aceto-orcein staining, as described elsewhere^40^. Oocytes with a polar body were defined as mature.

### Evaluation of developmental competence

After IVM, some OCGCs were subjected to IVF as previously described^41^. Briefly, after thawing frozen semen from a Holstein bull in a 37 °C water bath for 40 s, motile sperm (2 × 10^6^ sperm/mL) were separated by centrifuging twice (300×*g*) for 5 min in a semen preparation medium (BO-SemenPrep; IVF Bioscience, Cornwall, UK). OCGCs were cultured in a group with separated sperm in 500 µL of fertilization medium (BO-IVF; IVF Bioscience) in 4-well dishes (9–15 zygotes/well) for 18 h at 38.5 ℃ in 5% CO_2_ in air. After IVF, the presumptive zygotes were cultured in a 30 µL droplet (9–15 zygotes/droplet) of culture medium (BO-IVC; IVF Bioscience) with an overlay of paraffin oil at 5% O_2_, 5% CO_2_, and 90% N_2_ at 39 ℃ for 150 h. The cleavage and blastocyst rates were examined at 48 h and 168 h after IVF, respectively. Cell numbers in the blastocysts were counted as previously described elsewhere^42^.

### E_2_ and P_4_ assays of IVG culture medium

Spent medium from IVG was assayed for the E_2_ and P_4_ concentrations using a competitive double-antibody enzyme immunoassay as described previously^26^. The assay sensitivities were 7.1 pg/well for E_2_ and 11.2 pg/well for P_4_. The inter- and intra-coefficients of variation were 5.6 and 4.0% for E_2_ and 3.4 and 3.9% for P_4_, respectively. The steroid hormone production during each period (days 0–4, 4–8, and 8–12) was calculated as previously described^32^.

### Detection of ROS and GSH in oocytes

Briefly, denuded oocytes were washed with Dulbecco’s PBS (DPBS) (Nissui Pharmaceutical CO., LTD. Tokyo, Japan) supplemented with 0.2% PVP (DPBS-PVP) and treated with 10 µM 2ʹ,7ʹ-dichlorodihydrofluorescein diacetate (DCHFDA) for ROS staining, or 10 µM CellTracker Blue (Thermo Fisher Scientific Inc. Waltham, USA) for GSH staining at 38.0 ℃ for 30 min. The denuded oocytes were then washed three times with 0.2% DPBS-PVP, and placed on a glass slide. Images of the oocytes were acquired using a digital fluorescence microscope (BZ-9000; Keyence, Osaka, Japan). The fluorescence intensity (arbitrary unit) was calculated using Image J software. The mean fluorescence intensity of the control group (experiment 2) or the cysteine untreated group (experiment 3) in each replicate was set at 1.0, and that of the heat shock group or the cysteine treated group was expressed as a relative value, as previously described^43^.

### Evaluation of the transzonal projection (TZP) number

The OCGCs were washed in DPBS containing 0.1% PVA (DPBS-PVA), then fixed in 4% paraformaldehyde in DPBS-PVA for 60 min. Fixed OCGCs were washed in DPBS-PVA, and the oocytes were denuded with a fine pipette. The denuded oocytes were then stored in DPBS-PVA containing 1 mg/mL bovine serum albumin (DPBS-PVA-BSA) at 4 ℃ overnight. The oocytes were treated with fluorescein isothiocyanate-labeled Phalloidin (2 mg/mL in DPBS–PVA–BSA; Phalloidin–fluorescein isothiocyanate) at 38.5 ℃ for 90 min. The oocytes were washed in DPBS–PVA–BSA before being mounted on glass slides and observed under a confocal laser scanning microscope (LSM800) with ZEN software (Carl Zeiss, Jena, Germany). The pictures of oocytes were visualized using software (NIS-Elements D 4.10.00, Nikon, Tokyo, Japan). The pictures of the widest cross-section of each oocyte were selected for counting TZPs. In each picture of an oocyte, the TZPs completely crossed/traversing the zona pellucida from the cumulus cells to the oocytes were confirmed visually and counted one by one using a counter tool of the software manually.

### Experimental design

A schematic drawing of the experimental design is shown in Fig. 1b. The present study was conducted from June 2020 to February 2022 in Hokkaido, Japan. Bovine ovaries were collected from a slaughter house: slaughterhouse 1 (Hokkaido Hayakita meat inspection center, Abira, Hokkaido; 42° 43′ 30ʹʹ N, 141° 46′ 40ʹʹ E) for experiment 1 and 2, and from two slaughterhouses: slaughterhouse 1 and slaughterhouse 2 (NICHIRO CHIKUSAN CO., LTD., Nayoro Plant, Nayoro, Hokkaido; 44° 22′ 29ʹʹ N, 142° 27′ 39ʹʹ E) for experiment 3. The mean monthly ambient temperature (AT) ranged from − 7.5 to 21.6 ℃ in the location of slaughterhouse 1, and − 9.6 to 23.2 ℃ in that of slaughterhouse 2, respectively. To exclude bias among the sampling timings and between each slaughter house, we examined two groups every time in each replicate. In each experiment, OCGCs were evenly distributed to the control and the heat shock groups (experiment 1 and 2), or the cysteine untreated and the cysteine treated groups (experiment 3) according to the oocyte diameter and the thickness of the mural granulosa-cell layer.

During IVG culture, OCGCs in the heat shock group (experiment 1 and 2) and in both groups (experiment 3) were cultured at a range of temperatures (38.5 °C for 5 h, 39.5 °C for 5 h, 40.5 °C for 5 h, and 39.5 °C for 9 h) that were similar to those experienced by heat-stressed cows (Fig. 1a)^29^. To obtain the temperature condition, we changed the temperature setting of the incubator. OCGCs in the control group were cultured at a constant temperature of 38.5 °C for 24 h, mimicking the body temperature of cows without heat stress^28^.

#### Experiment 1: effects of heat exposure during IVG culture on OCGC morphology, oocyte growth, and maturational and oocyte developmental competence

A total of 1182 OCGCs were cultured for experiment 1 and 2 with the following sample distribution: 590 OCGCs in the control group and 592 OCGCs in the heat shock group. The viability of OCGCs was calculated based on 689 OCGCs that were cultured for 12 days (345 from the control group and 344 from the heat shock group). The percentage of antrum formation in the granulosa cell layer was calculated based on 406 OCGCs surviving on day 12 (208 from the control group and 198 from the heat shock group). Both the viability and antrum formation of the OCGCs were evaluated at days 4, 8 and 12 of IVG culture. Some surviving OCGCs (58 from the control group and 54 from the heat shock group) after 12 days of IVG culture were subjected to IVM culture to check the nuclear status of the oocytes. The diameters of these oocytes before IVG culture and after IVG and IVM cultures were also measured. Some surviving OCGCs (47 from the control group and 48 from the heat shock group) were subjected to IVM, IVF, and IVC to investigate their developmental competence. OCGCs cultured until IVM (evaluation of growth and nuclear status) or IVC (evaluation of developmental competence) were derived from different culture sessions. The blastocyst rates were calculated based on the number of inseminated oocytes.

#### Experiment 2: effects of heat exposure during IVG culture on the steroidogenesis of granulosa cells, the ROS and GSH levels in oocytes, and the number of TZPs in oocytes

The production levels of E_2_ and P_4_ in the culture medium were assessed every 4 days (days 4, 8, and 12). Spent medium was derived from the IVG culture of 33 OCGCs in the control group and 31 OCGCs in the heat shock group. The intracellular ROS and GSH levels of the oocytes were evaluated every 4 days during IVG culture. For the ROS assay, 141 OCGCs were used (71 from the control group and 70 from the heat shock group). For the GSH assay, 177 OCGCs were used (85 from the control group and 92 from the heat shock group). The number of TZPs was evaluated at days 0, 4, 8, and 12 of IVG culture. To evaluate the number of TZPs, 174 OCGCs were used (76 from the control group, 73 from the heat shock group and the remaining 25 OCGCs were used to evaluate the number of TZPs at day 0).

#### Experiment 3: effects of cysteine supplementation on the developmental competence and GSH levels of oocytes exposed to high temperature during IVG culture

A total of 257 OCGCs were cultured for experiment 3 with the following sample distribution: 130 OCGCs in the cysteine untreated group (IVG medium supplemented with 0.0 mM cysteine) and 127 OCGCs in the cysteine treated group (IVG medium supplemented with 1.2 mM cysteine). The cysteine concentration of medium in the cysteine untreated group was not 0.0 mM exactly, since the basal medium (HEPES-buffered TCM-199) contains about 0.6 µM cysteine. OCGCs in both groups were cultured in a range of temperatures (38.5 °C for 5 h, 39.5 °C for 5 h, 40.5 °C for 5 h, and 39.5 °C for 9 h) that were similar to those experienced by heat-stressed cows^29^. Some surviving OCGCs (49 from the cysteine untreated group and 43 from the cysteine treated group) after 12 days of IVG culture were subjected to IVM, IVF and IVC to investigate their developmental competence. Other surviving OCGCs (18 from the cysteine untreated group and 19 from the cysteine treated group) after 12 days of IVG culture were used to evaluate the GSH levels in oocytes.

### Statistical analysis

All statistical analyses were performed using software (JMP version 14.0.0, SAS Institute, Cary, USA). The normality of distribution was analyzed by the Shapiro–Wilk W test, and the homogeneity of variance was analyzed by the Levene test for all data. The diameters of the oocytes were evaluated by two-way ANOVA (group × culture period) followed by the Tukey–Kramer test. The viability and antrum formation of OCGCs, and the nuclear maturation and cleavage rates were analyzed by the chi-squared test, while the blastocyst rate was analyzed by Fisher’s exact test. As some data for the E_2_ and P_4_ production and the number of TZPs were non-parametric, the E_2_ and P_4_ production levels and the number of TZPs were analyzed by the Kruskal–Wallis test followed by the Steel–Dwass test. The intracellular ROS and GSH levels of the oocytes and the pH value of the IVG medium between the groups on the same culture day were analyzed by the Student’s *t*-test. The cell numbers in the blastocysts between the groups in experiment 3 were analyzed by the Mann–Whitney U test. P-values of less than 0.05 were considered significant.

## Data Availability

All the relevant data are included in the manuscript.
